# Real-Time Queue Length Detection with Roadside LiDAR Data

**DOI:** 10.3390/s20082342

**Published:** 2020-04-20

**Authors:** Jianqing Wu, Hao Xu, Yongsheng Zhang, Yuan Tian, Xiuguang Song

**Affiliations:** 1 School of Qilu Transportation, Shandong University, Jinan 250061, China; jianqingwusdu@sdu.edu.cn; 2 Department of Civil and Environmental Engineering, University of Nevada, Reno, NV 89557, USA; haox@unr.edu (H.X.); yongshengz@unr.edu (Y.Z.); yuantian@nevada.unr.edu (Y.T.)

**Keywords:** queue length, roadside sensor, vehicle detection

## Abstract

Real-time queue length information is an important input for many traffic applications. This paper presents a novel method for real-time queue length detection with roadside LiDAR data. Vehicles on the road were continuously tracked with the LiDAR data processing procedures (including background filtering, point clustering, object classification, lane identification and object association). A detailed method to identify the vehicle at the end of the queue considering the occlusion issue and package loss issue was documented in this study. The proposed method can provide real-time queue length information. The performance of the proposed queue length detection method was evaluated with the ground-truth data collected from three sites in Reno, Nevada. Results show the proposed method can achieve an average of 98% accuracy at the six investigated sites. The errors in the queue length detection were also diagnosed.

## 1. Introduction

Queue length has been used in many transportation areas, including but not limited to performance evaluation at signalized intersections, adaptive signal control, adaptive ramp metering and travel route selection [1]. Some applications such as the optimal signal control and travel route selection require real-time queue length information [2]. Queue length can either be estimated or directly detected. The typical queue estimation methods include the input-output method and the shockwave method. The input-output method uses advanced detector actuation, parametric data (headway, storage capacity, etc.) and phase change information (for signalized intersections) to estimate the queue length. The input-output method has the assumptions that the vehicles stay in the same lane after passing the advanced detector and the vehicles follow the first-in-first-out (FIFO) principle [3]. However, the accuracy of the input-output approach is limited by the detector’s counting error [4]. Lee et al. proposed a singular-point correction method to eliminate the accumulated counting error over time [5], but the singular-point correction method required the proper calibration based on the different features of field data, which limited the transferability of the method. Liu et al. [6] applied Lighthill–Whitham–Richards (LWR) shockwave theory to estimate queue length with the high-resolution traffic signal data. A detailed method of identifying break points was documented in their paper. The testing results showed that their method was able to estimate long queues with relatively high accuracy. However, their proposed model could not estimate the queue length under oversaturation. Using probe trajectory data for queue length estimation has been another hot topic for transportation researchers [7]. Cheng et al. [8] developed a cycle-by-cycle queue length estimation method with sampled vehicle trajectory data. Queue length was estimated based on the LWR shockwave theory. Later Hao et al. [9] developed a Bayesian Network (BN)-based method for cycle-by-cycle queue length estimation at signalized intersections. The travel times collected from mobile traffic sensors were the input data. The results showed that the BN-based method has a better performance than the method proposed in Cheng et al. [8]. Cai et al. [10] used fusing data from point and mobile sensors to estimate queue length at signalized intersections with the shockwave theory. This method assumed that at least one queue vehicle can be obtained in one cycle. Their model could not properly estimate the queue length when the arrival flow was unstable. The probe trajectory-based approaches suffer a major limitation: the sample rate can influence the accuracy of the queue length estimation [11].

Recent studies showed an increasing interest for queue length estimation using connected vehicle (CV) technology. Li et al. [12] used the probe trajectory and signal timing data extracted from the CV network to estimate real-time queue length with an event-based method. That paper used a lot of default values in the calculation (e.g., headway is 2.5 s, constant deceleration rate is 1.55 m/s^2^). Those default values may not reflect the different drivers’ driving behavior. Christofa et al. [13] developed two methods: gap-based method and shockwave-based method for queue spillback detection. The results showed that this approach can detect the occurrence of spillbacks for a range of penetration rates. Tiaprasert et al. [14] applied a discrete wavelet transform (DWT) for queue estimation using CV technology. One of the assumptions for the DWT is that the penetration ratio is known. For real situation, the detailed penetration ratio information may not be available. Yang and Menendez [15] proposed a convex optimization-based method for queue length estimation in a CV network. The validation showed that the estimation error went larger for oversaturated scenarios. The challenge in those methods using CV technology was the low penetration rate of CV on the roads, and the penetration rate of CV is expected to be low in the near future [16].

Though the queue length can be estimated—and the accuracy can be relatively high—those methods usually have their own assumptions, indicating that the methods may only work for some specific locations. Other than queue length estimation, researchers are also looking for approaches to directly detect the queue length. The image-based method can be an option for queue length detection [17]. Siyal and Fathy [18] applied the neural network to extract queue length from the image extracted from cameras. However, the computational load of this method was high since the neural network was applied. Cai et al. [19] used the texture difference and edge information to detect the vehicles and queue length from the videos. The practice showed that the inferences attached to vehicles and marks on the road can impact the accuracy of the queue length detection. Satzoda et al. [20] used the edges and dark features in the image for queue length detection. The evaluation showed that nearly 100% accuracy can be achieved in their testing database. The limitation of this method was that a lot of calibrations were required for driving detection zones in the images. The performance of the camera can also be greatly influenced by the light conditions. Xu et al. [21] developed a method for queue length detection by vehicle-to-RSU (V2R) communication. However, this approach was based on the assumption that all vehicles were installed with a communication system and a global positioning system (GPS). As a result, this method was also limited by the low penetration rate of CV on the roads.

The roadside LiDAR provides a solution for queue length detection regardless of whether vehicles can communicate with each other on the roads. The 360-degree LiDAR can scan all the objects in its detection range [22]. This means the penetration ratio of CV has no influence on the queue length detection using the roadside LiDAR. Furthermore, queue length can be detected using the LiDAR with or without the traffic signal information. Those advantages of LiDAR make the real-time queue length detection possible. This paper developed a systematic procedure for queue length detection with roadside LiDAR. The rest of the paper is structured as follows. Section 2 introduces the vehicle detection algorithm with roadside LiDAR data. The algorithm of queue length detection is documented in Section 3. Section 4 evaluates the proposed method using real-world collected LiDAR data. The last section summarizes the major contribution of this paper.

## 2. Materials and Preprocessing

The roadside LiDAR refers to the LiDAR deployed in a stationary location along the roadside. The roadside LiDAR (usually rotating LiDAR) has lower resolution and a lower price compared to the airborne LiDAR and on-board LiDAR (mobile LiDAR) considering the massive deployment in the near future. This paper used two types of LiDAR: VLP-16 and VLP-32c for data collection. For the detailed parameters of VLP-16 and VLP-32c, we refer the readers to [23].

But in theory, our proposed queue length detection method can work for any brand of rotating LiDAR after necessary calibration based on the different setting parameters of the LiDAR.

The roadside LiDAR can be installed permanently (on the top of a pedestrian signal) or temporarily (on a tripod) for data collection [24]. The recommended height for LiDAR installation is 2–3 m above the ground to avoid possible man-made destruction and to reduce occlusion issues considering the limited vertical field of view [25]. The scanning rate of the LiDAR is set as 10 Hz. The proposed vehicle detection procedure includes five major steps: background filtering [26], point clustering [27], object classification [28,29], lane identification [30,31] and object association [32]. Vehicle trajectories can be generated with the proposed method.

### 2.1. Background Filtering

**Figure 1:**
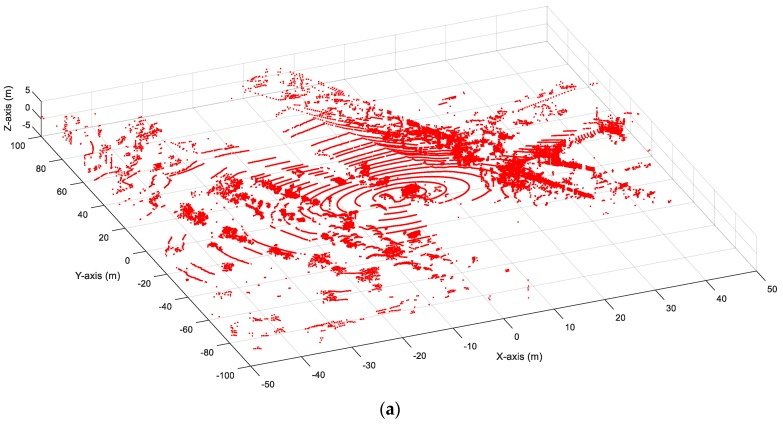

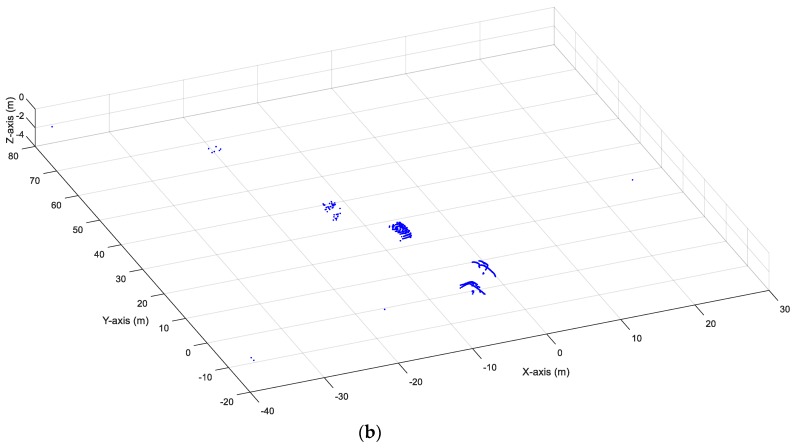
Before-and-after background filtering: (**a**) before background filtering; (**b**) after background filtering.

For queue length detection, the objects of interest are the vehicles on the road. Background filtering is used to exclude the other irrelevant information (buildings, trees and ground points) and to keep the moving objects (vehicles, pedestrians and other road users) in the space at the same time. This paper applied a point density-based unsupervised algorithm named 3D-DSF developed by Wu et al. [25] for background filtering. The 3D-DSF first integrated the data collected in a time period (such as 5 min of data) into one space based on the XYZ coordinates of the LiDAR points. The whole space was then rasterized into small cubes with the same side length (0.1 m was used as the side length considering the accuracy and the computation load) [26]. The point density of the cubes representing the background should be higher than that of the cubes representing the moving objects after frame aggregation. By giving a pre-defined threshold of point density, the location of the cubes representing background can be then identified and stored in a 3D array. An automatic threshold identification method was well documented in the reference [26]. Any point located in the 3D array was then excluded from the space. Figure 1 shows an example of before-and-after background filtering. The previous studies [33,34,35] showed that 3D-DSF can exclude more than 95% of background points from the raw LiDAR data.

### 2.2. Point Clustering

The points in the LiDAR data are stored disorderly, indicating that the points representing the same object are not grouped together. Point clustering is used to find the points belonging to the same object and to provide the same ID to those points. Another function of object clustering is to exclude the noises left after background filtering since not all background points can be filtered with 3D-DSF. This paper applied a revised density-based spatial clustering of applications with noises (DBSCAN) for object clustering [36]. DBSCAN defined one cluster as a set of points with high density. Compared to the widely used K-means clustering, DBSCAN does not need to know the number of clusters in advance and can find any shape of the clusters in the data [27]. There are two initial parameters for DBSCAN: searching radius (eps) and minimum containing points (minPts). DBSCAN starts with a random unvisited point A and marks the points with a distance ≤ eps from point A as the neighbors of point A. The following criteria are applied.

- If the number of the neighbors of point A ≥ minPts, then point A and its neighbors are marked as a cluster and point A is marked as a visited point. DBSCAN then uses the same method to process the points of other unvisited points in the same cluster to extend the range of the cluster.
- If the number of the neighbors of point A < minPts, then point A will be marked as a noising point and a visited point.

**Figure 2:**
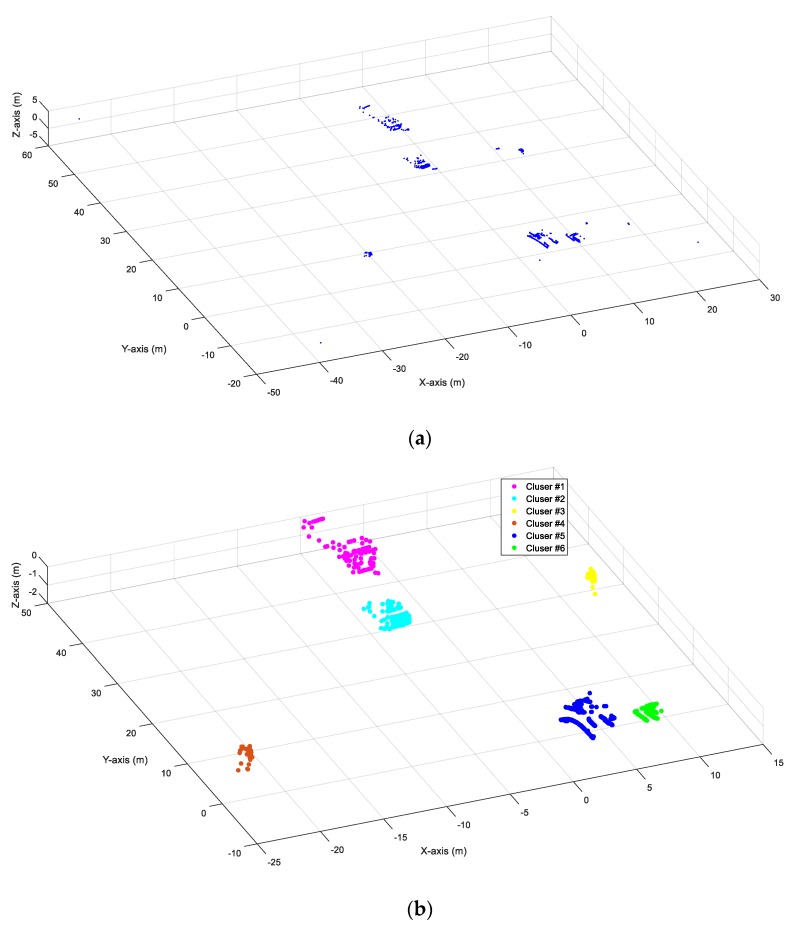
Before-and-after point clustering: (**a**) before point clustering; (**b**) after point clustering.

Using the above-mentioned criteria, DBSCAN can process all those unvisited points. However, one major disadvantage of DBSCAN is that it could not cluster the points with uneven density effectively. For LiDAR data, the number of points representing the same object decreased with the increasing distance to the LiDAR, indicating the point density changes as the distance to the LiDAR increasing. To fix this issue, a revised DBSCAN with adaptive parameters are applied for point clustering. Different eps and minPts were applied for the points based on the distance from the LiDAR and the mechanical structure (field of view, angular resolution and the distance between two adjacent beams) of the LiDAR. The detailed calculation of eps and minPts was documented in our previous research [27]. The accuracy of the revised DBSCAN is 96.5% in average. Figure 2 shows an example of before-and-after point clustering. It is shown that the revised DBSCAN algorithm can successfully identify six objects and exclude the noise from the LiDAR data in Figure 2.

### 2.3. Object Classification

**Figure 3:**
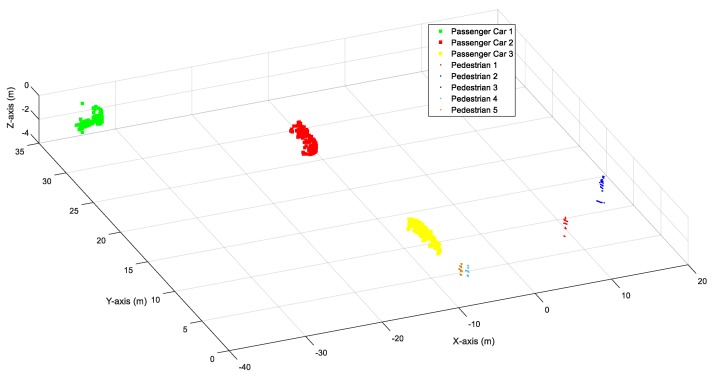
Vehicle classification.

There may be different types of road users (vehicles, pedestrians and bicycles) on the road. It is then necessary to distinguish the vehicles from other types of road users for queue length detection. This paper aims to classify the objects into one of the four classes (passenger car/pickup, trucks/bus, pedestrians and bicycles). Six features (object length, object height, the difference between object height and object length, distance to LiDAR, number of points and object height profile) extracted from the point cloud were used to represent the difference between different classes. A random forest (RF) classifier was trained for object classification. The RF is a supervised algorithm that aggregates multiple decision trees. Our previous study [28] compared the performance of different methods (k-nearest neighbor, Naïve Bayes, RF and support vector machine) for roadside LiDAR classification. It was shown that RF can provide the best accuracy among those investigated methods. This paper used the public database [28] collected by the Center for Advanced Transportation Education and Research (CATER) in University of Nevada, Reno to train the RF classifier. The testing results showed that the RF can achieve an overall 95.3% accuracy for object classification. Figure 3 shows an example of the results of object classification. The RF classifier can correctly identify three passenger cars and five pedestrians in the point cloud.

### 2.4. Lane Identification

The lane information is important for lane-based queue length detection. It is assumed that the probability of the vehicles changing lanes near the intersection is low. This means the density of vehicle points on each lane is higher than that of vehicle points near the boundary area of the lane. We applied a revised grid-based clustering (RGBC) developed by the authors’ team for lane identification [31]. The RGBC first integrates the vehicle points from multiple frames into one space. The whole space is segmented using a hierarchical segmentation to identify road areas and non-road areas. The whole space is divided into squares with a relatively larger side length, such as 10 m. This level is named as the first level. Each square will be identified as a road area and a non-road area based on the number of points in it. If there is no point in the square, this area will be identified as a non-road area. If there is at least one vehicle point in the square, this area will be considered as a road area. For the square considered as a road area, it will be further divided into a children level with a smaller side length. The same procedure will be used to judge whether each square represents the road area or not. Step by step, a children level will be provided for each parent level until a bottom layer with a pre-defined side length (such as 0.1 m) is generated. For the squares in the bottom layer, a pre-defined threshold of point density will be provided to distinguish the squares representing road area and the squares representing the non-road area. For the road area, DBSCAN was applied to cluster the points in the same lane. It should be mentioned that the lane boundary identified here may not be the exact boundary of the lane, but the boundary of vehicle points in the same lane. The previous study showed that more than 96% of vehicles can be assigned to the correct lanes. The errors were mostly caused by lane-change vehicles. Another task in the lane identification is to detect the location of the stop line. In this paper, the location of the stop line was manually identified by checking the point intensity in the LiDAR visualization software Veloview. The point intensity of the stop line is higher compared to other objects [37]. A linear regression line can then be generated by linking two points at the boundaries of the stop line [38].

### 2.5. Object Association

The speed of the vehicle is crucial for determining the end of the queue [39]. The speed can be calculated based on the distance traveled by the vehicle in a time period. Therefore, it is necessary to track the same vehicle continuously at different frames. This research applied a global nearest neighbor (GNN) algorithm to link the point cloud representing the same vehicle at different frames. The GNN considers the vehicle in the current frame that has the minimum distance to the vehicle A in the last frame as vehicle A [40]. To calculate the speed, the algorithm used the point with the shortest distance to the LiDAR as a reference point for vehicle tracking. The speed (V) can then be calculated by:

(1) $$V=F*\sqrt{{\left(X_{i}-X_{i-1}\right)}^{2}+{\left(Y_{i}-Y_{i-1}\right)}^{2}+{\left(Z_{i}-Z_{i-1}\right)}^{2}}$$

where *XYZ* are the *XYZ* coordinates of the nearest point to the LiDAR, *i* is the frame ID and F is the rotating frequency of the LiDAR (unit: Hz). To evaluate the speed, we did a field test using a vehicle installed with a logger to extract the speed from the onboard diagnostics interface (OBD). The vehicle was also scanned by the roadside LiDAR and the speed was calculated. The OBD speed and the calculated speed were then compared. The testing results showed that about 98.8% of speed records calculated from GNN has a speed with a difference less than 2.4 km/h compared to the OBD speed [32].

## 3. Queue Length Detection

**Figure 4:**
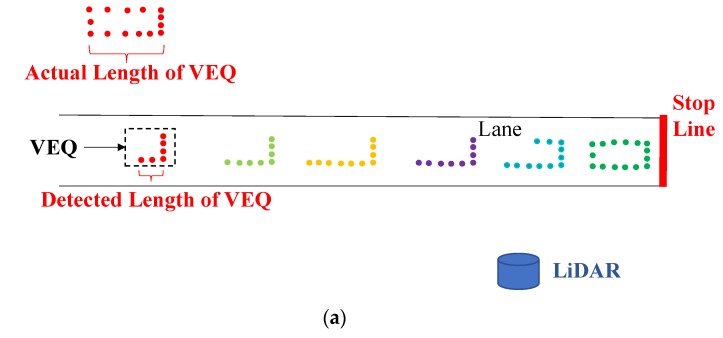

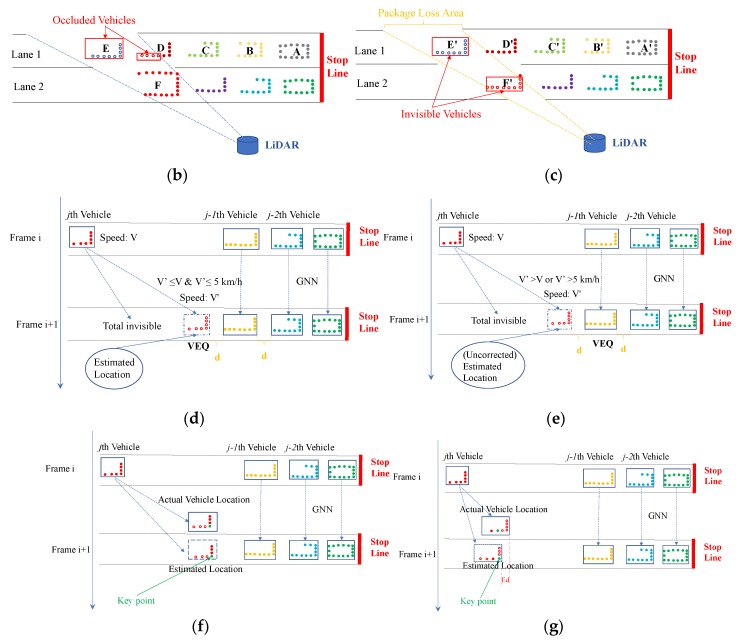
Queue length detection with occlusion and package loss issues: (**a**) occlusion issue, (**b**) occlusion issue, (**c**) package loss issue, (**d**) *VEQ* estimation: scenario 1, (**e**) *VEQ* estimation: scenario 2, (**f**) *VEQ* estimation: scenario 3, (**g**) *VEQ* estimation: scenario 4.

Inspired by the previous work [19,40], we assumed the following threshold to identify the end of a queue: if the speed of one vehicle is under 5 km/h, then this vehicle will be considered as in a queue; if the speed of one vehicle is equal to or higher than 5 km/h, this vehicle would not be considered as in a queue. The vehicle at the end of the queue (*VEQ*) is the key vehicle to determine the length of a queue. Since the *VEQ* may be relatively far away from the LiDAR if the road is congested, the length of the vehicle at the end of the queue (*LVEQ*) may not be fully detected (point density decreases with the increasing distance from the LiDAR) [41], as shown in Figure 4a. Therefore, it is necessary to estimate the LVEQ. We used a simple rule-based method for *LVEQ* estimation. We assumed that the average length of a passenger car is 6 m [42]. Therefore, if the detected vehicle length (*DVL*) is longer than 6 m, we use the detected length as the vehicle length. If the detected vehicle length is shorter than 6 m, we used 6 m as the length of the vehicle. The strategy of the simple rule-based method can be expressed as:

(2) $$LVEQ=\{\begin{array}{c} DVL,\;if\;DVL\geq 6\;m\\ 6\;m,\;if\;DVL<6\;m \end{array}$$

Using Equation (2) can still have some errors in estimating the queue. But the influence of this error on the whole length of the queue should be very limited (We will present the result in the “Evaluation” part later).

If a truck or a larger vehicle is traveling in the lane close to the LiDAR, the vehicles on the other lanes may be blocked, which is called occlusion issue. The occlusion issue is a challenge to detect the end of the queue since the last one or several vehicles at the end of the queue may be blocked (invisible in the LiDAR). Figure 4b shows an example of occlusion issue. Vehicle E and part of vehicle D are invisible due to occlusion from vehicle F. Another challenge for detecting the end of the queue is the package loss issue. Package loss refers to the situation that some packages are lost due to the unstable connection between the LiDAR and the data storing device (usually a computer). As a result, there are a lot of sector-like areas which are invisible in the space. The package loss issue may also make the vehicle at the end of the queue invisible. An example of package loss issue is shown in Figure 4c. Vehicle E′ and most part of vehicle F′ are invisible since they are in the package loss area (sector-like area).

To fix those issues, the vehicle information in the past time (historical information) was used. The first task is to detect whether there is an occlusion issue or package loss issue. We assumed that drivers would slow down when they are approaching the queue, meaning the speed of the vehicle in the current frame should be less than or at least equal to the speed in the last frame. This assumption makes it possible to use the speed to estimate the location of the vehicle if the vehicle is invisible in the current frame. The following method was applied for *VEQ* estimation.

Assuming there are *j* vehicles traveling on the lane in frame *i*, the *j*th vehicle is the vehicle that farthest from the stop line. The speed of the *j*th vehicle is recorded as *V*. The *j*th vehicle in frame *i* + 1 can be total occluded, partial occluded or non-occluded. The following parts illustrated the *VEQ* identification method for three situations (total occluded, partial occluded and non-occluded).

In frame *i* + 1, if the *j*th vehicle is invisible (the ID of the *j*th vehicle in frame *i* could not be assigned to any vehicle in frame *i* + 1), then the location of the *j*th vehicle can first be assumed to be the end of the queue. The distance d between the *j*th vehicle and the *j* − 1th vehicle is directly copied from the distance between the *j* − 1th vehicle and the *j* − 2th vehicle. The speed of the *j*th vehicle can be then calculated as *V*′. If *V*′ ≤ *V* and *V*′ ≤ 5 km/h, the *j*th vehicle is considered as the *VEQ*, as shown in Figure 4d. If *V*′ > *V* or *V*′ > 5 km/h, the *j* − 1th vehicle is considered as the *VEQ*, as shown in Figure 4e. The algorithm can be illustrated as

(3) $$L=\{\begin{array}{cc} \overset{j}{\underset{1}{\sum }}l_{i},\;\; & if\;V_{\left(j+1\right)\mathrm{th}}^{\prime }\leq V_{j\mathrm{th}}\cap^{\;}V_{\left(j+1\right)\mathrm{th}}^{\prime }\leq 5\;\mathrm{km}/h\\ \overset{j-1}{\underset{1}{\sum }}l_{i},\;\; & if\;V_{\left(j+1\right)\mathrm{th}}^{\prime }>V_{j\mathrm{th}}\cup^{\;}V_{\left(j+1\right)\mathrm{th}}^{\prime }>5\;\mathrm{km}/h \end{array}$$

where $\overset{j}{\underset{1}{\sum }}l_{i}$ means the total length of the vehicles in frame *i*, $V_{j\mathrm{th}}$ is the vehicle of *j*th vehicle.

If the *j*th vehicle is visible in frame *i* + 1, the algorithm does not know whether the vehicle is partially occluded or non-occluded. The point of the *j*th vehicle that has the shortest distance to the LiDAR is selected as a key point. The key point is considered as the front corner of the *j*th vehicle. The length of the *j*th vehicle in frame *i* is used as the length of the *j*th vehicle in frame *i* + 1. If the front part of the *j*th vehicle is visible, then the key point can reflect the location of the *j*th vehicle correctly, as shown in Figure 4f. If the end part of the *j*th vehicle is visible, then the key point may be located at the middle of the length of the vehicle. As a result, there may be a distance error (Ed) between the estimated location and the actual location of the *j*th vehicle, as shown in Figure 4g. But Ed should be less than the length of the *j*th vehicle. The speed of the *j*th vehicle in frame *i* + 1 can then be calculated. The *VEQ* can be identified by checking the speeds of the vehicles in frame *i* + 1. The algorithm can be illustrated as

(4) $$L=\{\begin{array}{cc} \sum_{1}^{j}l_{i},\;\; & if\;V_{\left(j+1\right)\mathrm{th}}^{\prime }\leq V_{j\mathrm{th}}\cap^{\;}V_{\left(j+1\right)\mathrm{th}}^{\prime }\leq 5\;\mathrm{km}/h\\ \sum_{1}^{j-1}l_{i},\;\; & if\;V_{\left(j+1\right)\mathrm{th}}^{\prime }>V_{j\mathrm{th}}\cup^{\;}V_{\left(j+1\right)\mathrm{th}}^{\prime }>5\;\mathrm{km}/h \end{array}$$

where $\sum_{1}^{j}l_{i}$ means the total length of the vehicles in frame *i*, $V_{j\mathrm{th}}$ is the vehicle of *j*th vehicle.

It should be mentioned that due to the different locations of the vehicles in the LiDAR data, the vehicles in the queue (not *VEQ*) may also be occluded or partially occluded due to the package loss or occlusion issue. Those occluded vehicles do not influence the queue length detection as long as the *VEQ* can be detected/predicted.

## 4. Evaluation

The performance of the LiDAR was evaluated with the ground-truth data extracted from the camera and the raw LiDAR visualized in the open-source visualization software—Veloview. Two trained graduate students were hired to manually extract the queue length from the camera installed at the selected sites. The number of vehicles in the queue was recorded by checking the camera and the LiDAR data in Veloview. The length of the queue was calculated by checking the location of the reference in camera and in Google Earth. The results extracted by the two graduate students were considered as the ground-truth data. It should be mentioned that the ground-truth data are not the data with 100% accuracy due to the distance calculation error in Google Earth and the inevitable human error.

Four sites with different road features in Reno, Nevada were selected for evaluation. The features of the three sites are documented in Table 1.

**Figure 5:**
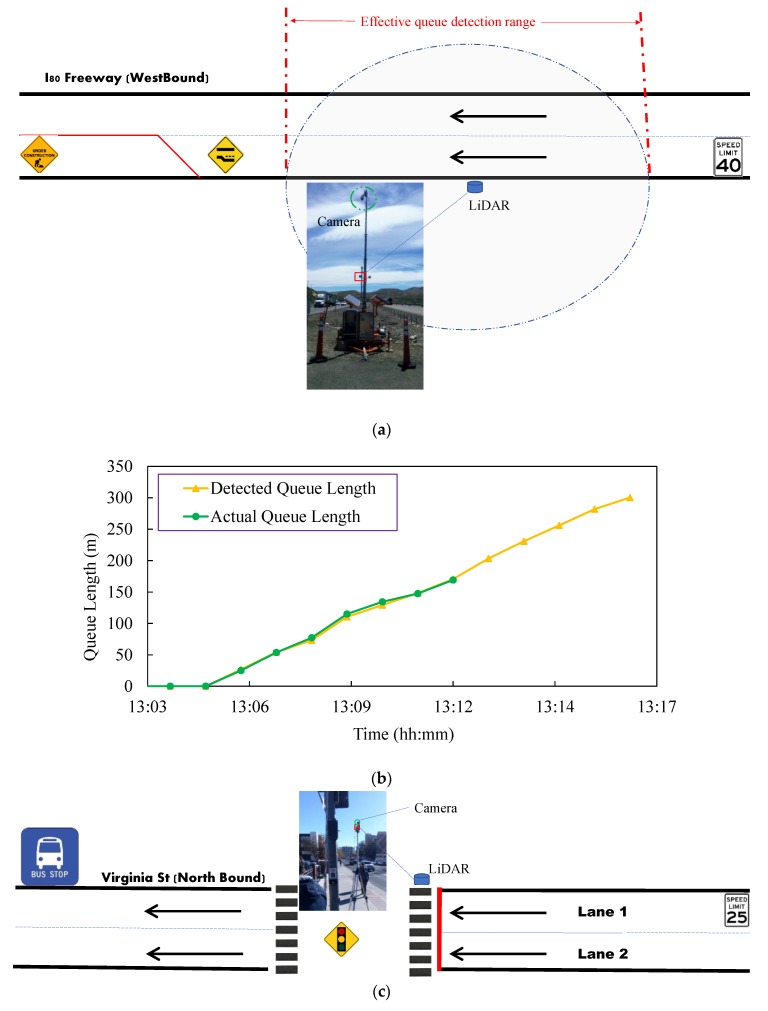

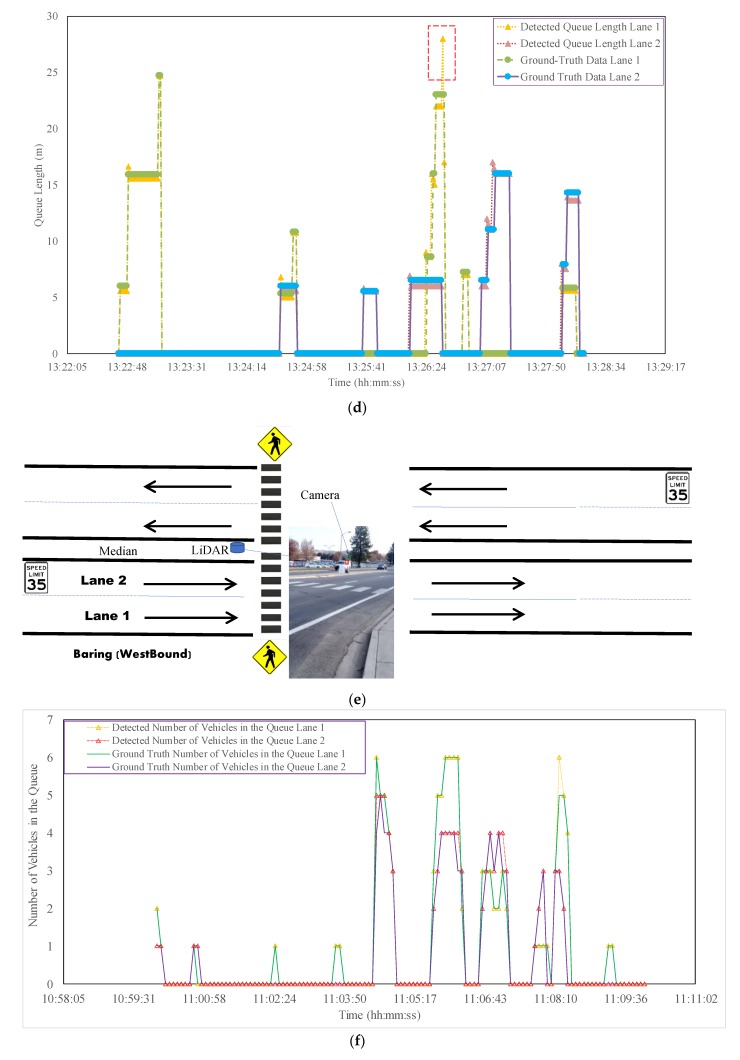
Evaluation of Queue Length Detection: (**a**) Site of I80 Westbound, (**b**) Results of Queue Length Detection at I80 Westbound, (**c**) Site of Virginia St @ Artemesia Way, (**d**) Results of Queue Length Detection at Virginia St @ Artemesia Way, (**e**) Site of Baring Blvd, (**f**) Results of Number of Vehicles in the Queue Detection at Baring Blvd.

Figure 5a shows the data collection location at the I80 work zone. Figure 5b shows the detected queue length and the measured queue length at the work zone in I80-W freeway. There are two lanes at the westbound and the left lane at the westbound was closed due to the pavement construction. It should be mentioned that there was not stop line near the work zone and the location of the LiDAR was not exactly located at the work zone since we did not get the permit to put LiDAR at the work zone. In other words, a zero value of queue length in Figure 5b does not really mean that there is no queue at the work zone, but no queue in the detection range of the LiDAR. The max queue length (among two lanes) in every minute was recorded by the proposed method and by checking the camera installed on top of the LiDAR. It is shown that from 13:05, the queue started to be formed. The camera can detect about a total of 169 m distance of the queue and the LiDAR can detect about a total of 300 m distance. The offsets between the queue lengths were manually extracted from the camera and the detected queue length from the LiDAR was small from 13:05 to 13:12. After 13:12, the queue length was difficult to be determined through the camera. Therefore, the green line in Figure 5b disappeared around 13:12. After 13:17, since the queue length was longer than the detection range of the LiDAR, the queue length could not be successfully detected. Therefore, the orange line disappeared around 13:17.

Figure 5c shows the data collection location at Virginia St @ Artemesia Way. The results of queue length detection are illustrated in Figure 5d. A total of six-minutes data were randomly selected for evaluation. The queue length for each lane was analyzed. It is shown that the detected queue length and the ground-truth queue length are close to each other for lane 2. However, there are some offsets between the detected queue length and the ground-truth queue length for lane 1 (as shown in the red rectangle in Figure 5d. Around 13:26, the detected queue length (28 m) was significantly higher than the ground-truth queue length (22 m). The longer detected queue length was caused by the definition of the end of the queue that the vehicle with the speed less than 5 km/h as the *VEQ*. But for ground-truth data, the queue length was identified by the graduate students based on their own judgment. Therefore, there were some offsets between the detected queue length and the ground-truth data. Figure 5e shows the data collection location at Baring Blvd. There is a pedestrian middle crossing at this site. Since there are not stop lines at this location, it is difficult to determine the start point of the queue. Therefore, we used the number of vehicles in the queue to represent the queue length. The results of the number of vehicles detection for each lane are illustrated in Figure 5f. It is shown that the detected number of vehicles and the ground-truth data matched very well though some errors existed in the detected results. There are two types of errors for detecting the number of vehicles. The first type of error can be seen at around 11:07 in Figure 5f. The detected number of vehicles in the queue was higher than the ground-truth number of vehicles in the queue in lane 2. By checking the camera data, it was found that the vehicle at the end of the queue was a commercial truck and it was chopped into two parts due to occlusion or package loss issue. As a result, the clustering algorithm clustered the truck as two vehicles. The second type of error can be seen at around 11:08 in Figure 5f. The detected number of vehicles in the queue was lower than the ground-truth number of vehicles in the queue in lane 1. The offsets were also caused by the different definitions of the end of the queue by the proposed algorithm and the graduate students.

Table 2 summarizes the distribution of the cumulative errors of the detected queue length and number of vehicles in the queue at the three sites. It is shown that 88.3% of calculated records had an error of less than 0.5 m in the queue length when compared to the ground-truth data and 96.2% of calculated records had an error of less than 3.0 m in the queue length when compared to the ground-truth data. As for the number of vehicles in the queue, 96.2% of the number of calculated vehicles in the queue was exactly matched to the human-counted records and 98.5% of calculated records had an offset within 1-vehicle count from the ground truth data. The maximum offset between the calculated records and the ground truth data were no more than 4 vehicles. The results indicated that the proposed method could achieve the relatively high accuracy for queue length detection.

## 5. Conclusions and Discussion

This paper presented a novel method for queue length detection using the roadside LiDAR data. Unlike the estimation methods used in most existing studies, the queue length can directly be detected with the proposed method. This proposed method can work for different road scenarios. The testing results showed that the proposed method can detect the queue length with high accuracy under different scenarios. The strategy of the proposed method is simple but effective in practice. With this proposed method, the accurate queue length can be provided in real-time for different applications. Therefore, the method is of great value in signal coordination especially in solving the initial queue estimation. Since the initial queue is unpredictable between cycle by cycle, this real-time measurement is proper to handle it. By capture the queue length, the proper offset can be calculated for each intersection along a corridor under each cycle to timely release the initial queue before the platoon arrives. This can be very helpful in adaptive signal control since the system is ready for real-time adjustment. Even for the traditional actuated-coordinated signals, the accurate trend of the initial queue change would help to decide the offset settings. On the other hand, it also directly supports the connected vehicle (which will avoid the queue blockage to the maximum extent) in the future. Vehicle to Vehicle or Vehicle to Infrastructure facilities can get the accurate queue information and in turn make adjustments from both vehicle approach and infrastructure approach with the least time loss in the process. The prospect of the method is bright. Queue length is also an important measure of effectiveness in the operational analysis. However, it is usually hard to estimate or measure; the problem has puzzled traffic engineers for a long time. The proposed method revolutionary decreases the measured time with the enhancement of the accuracy, which benefits a lot to operational analysis.

This paper did not compare the proposed queue length detection method with the existing queue length estimation methods since the data from the other sensors (such as loop detectors and signal timing) were not available. The future studies should also consider selecting one signalized intersection or a metered ramp to compare the proposed queue length detection method with other methods. For one LiDAR sensor, the longest detectable length of the queue is subject to the detection range of the LiDAR. If the queue length is out of the detection range, then another LiDAR to extend the detection range is needed. The ground-truth data were measured by two graduate students by checking the camera, Veloview and Google Earth. It is inevitable that errors exist in the ground-truth data in this paper. How to find a more accurate evaluation method for queue length detection is another research topic for future studies. Another limitation is that this paper used many assumptions (e.g., vehicle length is shorter than 6 m, vehicles with speed <5 km/h is considered in the queue), those assumptions can impact the accuracy of queue length detection. Future studies should consider to further improve the accuracy of queue length detection by reducing the assumptions of the proposed method. The performance of LiDAR can be reduced under adverse weather conditions, such as foggy, rainy, snowy weather. The proposed queue length detection highly relied on the accuracy of LiDAR detection. Therefore, future studies should also test the performance of the proposed queue length detection method under severe weather conditions.

## Figures and Tables

**Table 1:** Features of selected sites for evaluation.

Site	Speed Limit	Traffic Control	Areas for Queue Length Evaluation
I80 work zone	64.4 km/h (40 mph)	Left lane closed control	One westbound unclosed lane
Virginia St @ Artemesia Way	40.2 km/h (25 mph)	Signalized T-intersection	Two northbound through lanes
Baring Blvd at the front of the Reed High School	56.3 km/h (35 mph)	Yield sign for pedestrian crossing	Two westbound through lanes

**Table 2:** Cumulative errors of queue length detection.

Queue Length		Number of Vehicles in the Queue	
Error	Percentage (%)	Error	Percentage (%)
0–0.5 m	88.3	0 vehicle	96.2
0.5–1.0 m	89.8	1 vehicle	98.5
1.0–1.5 m	91.3	2 vehicles	99.1
1.5–2.0 m	94.5	3 vehicles	99.8
2.0–3.0 m	96.2	4 vehicles	100
